# Developing primary care teams prepared to improve quality: a mixed-methods evaluation and lessons learned from implementing a microsystems approach

**DOI:** 10.1186/s12913-018-3650-4

**Published:** 2018-11-09

**Authors:** Nancy Pandhi, Sally Kraft, Stephanie Berkson, Sarah Davis, Sandra Kamnetz, Steven Koslov, Elizabeth Trowbridge, William Caplan

**Affiliations:** 1 Department of Family and Community Medicine, University of New Mexico School of Medicine, MSC 09 5040, 1 University of New Mexico, Albuquerque, NM 87131 USA; 2 Population Health at Geisel School of Medicine at Dartmouth College, 1 Rope Ferry Rd, Hanover, NH 03755 USA; 3 Primary Care Academics Transforming Healthcare Collaborative, Madison, WI USA; 4 Planning and Business Development, UW Health, Madison, WI USA; 5 University of Wisconsin Law School, Madison, WI USA; 6 Center for Patient Partnerships, Madison, WI USA; 7 Department of Family Medicine and Community Health, University of Wisconsin School of Medicine and Public Health, Madison, WI USA; 8 Department of Pediatric and Adolescent Medicine, University of Wisconsin School of Medicine and Public Health, Madison, WI USA; 9 General Internal Medicine, University of Wisconsin School of Medicine and Public Health, Madison, WI USA

**Keywords:** Primary health care, Quality improvement, Patient care team, Microsystems

## Abstract

**Background:**

Health systems in the United States are increasingly required to become leaders in quality to compete successfully in a value-conscious purchasing market. Doing so involves developing effective clinical teams using approaches like the clinical microsystems framework. However, there has been limited assessment of this approach within United States primary care settings.

**Methods:**

This paper describes the implementation, mixed-methods evaluation results, and lessons learned from instituting a Microsystems approach across 6 years with 58 primary care teams at a large Midwestern academic health care system. The evaluation consisted of a longitudinal survey augmented by interviews and focus groups. Structured facilitated longitudinal discussions with leadership captured ongoing lessons learned. Quantitative analysis employed ordinal logistic regression and compared aggregate responses at 6-months and 12-months to those at the baseline period. Qualitative analysis used an immersion/crystallization approach.

**Results:**

Survey results (*N* = 204) indicated improved perceptions of: organizational support, team effectiveness and cohesion, meeting and quality improvement skills, and team communication. Thematic challenges from the qualitative data included: lack of time and coverage for participation, need for technical/technology support, perceived devaluation of improvement work, difficulty aggregating or spreading learnings, tensions between team and clinic level change, a part-time workforce, team instability and difficulties incorporating a data driven improvement approach.

**Conclusions:**

These findings suggest that a microsystems approach is valuable for building team relationships and quality improvement skills but is challenged in a large, diverse academic primary care context. They additionally suggest that primary care transformation will require purposeful changes implemented across the micro to macro-level including but not only focused on quality improvement training for microsystem teams.

## Background

In the United States, health systems are increasingly required to become leaders in quality improvement (QI) in order to compete successfully in a value-conscious purchasing market [1–3] Effective clinical teams are considered essential to the production of high-value systems of care particularly within primary care. The clinical microsystems framework [4] is one approach to training primary care teams how to engage in QI activities. Based on learnings from analyses of high-performing service organizations both external to and within health care [4, 5], this approach recognizes that health care value is created (or lost) by front-line teams informed by the needs of their patients and capable of designing and implementing change. In addition to disciplined process improvement education, the Microsystem approach recognizes the importance of managing relationships between microsystem members and between microsystems and the larger macro-systems [4]. Established curriculum include training on practice assessment, process improvement, socio-cultural and organizational change skills [6].

Despite widespread use of the Microsystems approach across multiple countries and hospital and ambulatory care settings [7–11], published evaluations of its implementation within United States primary care settings are limited in number and scope. This gap is surprising given an international emphasis on re-engineering primary care [12–16], and this approach being considered as one of the first few comprehensive models, besides the Chronic Care Model [17], the and the Idealized Design of Clinical Office Practices [18], that address team-based primary care redesign [19]. Two extensive qualitative evaluations have been conducted outside of the United States. These describe higher staff morale, empowerment, commitment, and clarity of purpose resulting from this approach [20, 21]. A single U.S.-based article briefly describes inclusion of microsystems training as part of a collaborative primary care faculty development initiative, and suggests that it would do best when there is “shared accountability for relationship service and reliability across the three [primary care] disciplines.” [22] Given the unique characteristics of primary care in an academic setting (e.g., providers who perform clinical care part-time among other responsibilities and the presence of trainees), further understanding of the strengths and limitations of the Microsystems approach is needed.

From 2008 to 2014, a Microsystems approach was implemented with 58 primary care teams at a large Midwestern academic health care center that was aligning its primary care disciplines and embarking upon an ambitious primary care delivery system redesign. This study was designed to address the following questions: 1) what was the 6 and 12 month impact of the Microsystems approach on primary care team member perception of effectiveness and cohesion, quality improvement skills, and communication skills? 2) What challenges occurred during program implementation and were these able to be overcome? Given the importance of context in understanding quality improvement interventions, we also provide a detailed description of the delivery system and intervention in hopes of informing others embarking on similar system-wide multi-clinic primary care improvement efforts.

## Methods

### Description of the delivery system

At the launch of the redesign initiative in 2008, the academic health center consisted of three separate, legal organizations: UW School of Medicine and Public Health, UW Medical Foundation, and UW Hospital and Clinics. Clinics were owned and operated by one of these organizations but were collectively branded as UW Health. Forty-six primary care clinics were located throughout the state of Wisconsin, with the majority in the Madison area. There were 276 primary care physicians: 38 pediatricians, 62 general internists, and 156 family physicians who conducted ~ 649,000 primary care visits annually. Practices varied in size from 1 to 20 physicians.

### The impetus for redesign

The three chief executives launched the UW Health Primary Care Redesign initiative, an ambitious effort to transform delivery of primary care within the entire system regardless of clinic location, primary care specialty or ownership [23]. The need to radically transform care delivery arose from access issues within primary care and lagging clinical quality performance compared to other health systems in the state as publicly reported by the Wisconsin Collaborative for Healthcare Quality, a voluntary performance reporting system (www.wchq.org). These issues reached a critical state when a new provider group entered the local market and successfully recruited several faculty physicians. This acute loss of established physicians coupled with the national difficulty of attracting new physicians to primary care created a short term access crisis that prompted a focus on transforming the primary care system to achieve what is now termed the quadruple aim [24].

### Guiding principles and vision

The initiative was guided by evidence-based principles for transformative change [25–27]. UW Health leaders in quality and safety had developed a framework for change based on redesign principles from the Institute of Medicine (IOM) and organizational behavior theory that was used to organize its large-scale change initiatives [28]. Kotter’s 8-steps for organizational transformation provided guidance for change over time [25]. A governance structure, the Primary Care Steering Committee, was created consisting of physicians and administrative leaders responsible for primary care. A vision for the transformed primary care system was created by a broad representation of front-line clinicians and staff working collaboratively with senior leadership and communicated widely throughout the organization. The academic health system’s Health Innovation Program was created to integrate healthcare research and practice. In support of this mission, health services researchers were included on the Initiative’s strategy oversight and implementation teams. Researchers led the study design for the evaluation including collection of qualitative data that was rapidly analyzed and shared with operational leaders in ‘near real time,’ providing important findings that informed future implementation strategies.

### The microsystems approach

The Dartmouth Clinical Microsystem curriculum was considered by the Steering Committee to be the framework that best fit existing organizational strengths in process improvement education and its readiness to address change at all levels of the health system. Prior to implementation of this approach, primary care clinic leaders had participated in structured process improvement education incorporating concepts from various models including the Institute for Healthcare Improvement (IHI) model of improvement, systems thinking, and Lean Six Sigma concepts [29–31]. The clinical microsystem approach specifically was selected because it allowed for those who were affected by the change to design the change by equipping front-line care team members and administrative managers with common team-building and improvement skills useful for redesigning care processes.

**Fig. 1:**
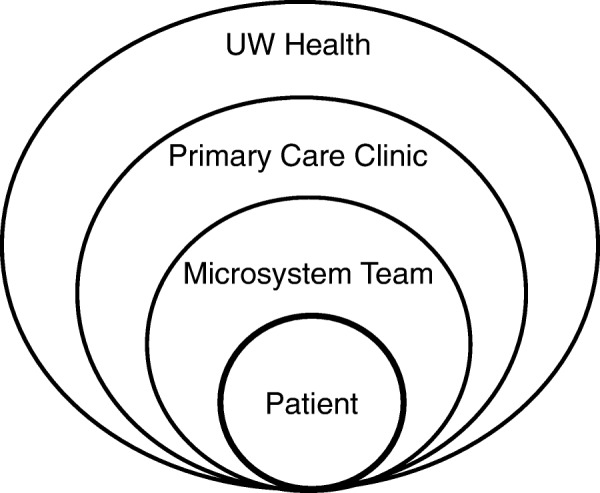
Organizational redesign levels

In this approach, a microsystem is defined as a small care unit consisting of a care team, its panel of patients, and the core processes that produce its patterns and norms [4]. Within the organization, a primary-care microsystem generally consisted of a physician (or several part-time physicians), a receptionist, registered nurse and other clinical staff (e.g. medical assistant, licensed practical nurse) and often included an advanced practice provider and residents. Figure 1 depicts these organizational levels.

### The microsystems program

Table 1 provides a summary of program goals, components and involvement. As shown, program activities consisted cohort learning sessions, team meetings, and practice coaching. During these activities teams learned how to continuously assess their microsystems. Data analysis and team input drove the establishment of improvement aims and implementation of interventions. Initially aims could be developed in any area, but by the third cohort microsystem teams were asked to focus improvement efforts on specific areas (e.g. access) that were aligned with organizational priorities and supported by organizational resources.

**Table 1:** Microsystems program goals, components, and people involved

People involved	Goals	Components
Primary care steering committee Quality Safety and Innovation Department leadership Program managers and coaches Microsystems evaluation team	Lead, implement and evaluate the program	Discuss strategy
		Review implementation details
		Design evaluation
		Collect data, analyze and report back
Primary care and quality Safety and innovation leaders Practice Coaches Microsystem teams	Build quality improvement knowledge and skills and teamwork competences	3–4 half day cohort learning sessions
		Weekly team meetings with practice coach
		Follow the FOCUS (Find-Organize-Clarify-Understand-Select) PDCA (Plan-Do-Check-Act) improvement process
		Role rotation (e.g facilitator, time keeper, note taker)

Coaches provided ongoing teaching on how to follow the FOCUS (Find-Organize-Clarify-Understand-Select) PDCA (Plan-Do-Check-Act) improvement process and apply appropriate improvement tools. The coach also helped facilitate team building, team communication and patient engagement. After 6 months, coaches met their teams with decreasing frequency to reinforce skills and by 12 months transitioned contact to an as-needed basis.

### Program evaluation design

The overall objectives of this concurrent mixed-methods evaluation was to understand: 1) the short term and sustained impact of the program on team effectiveness and cohesion, quality improvement skills, and communication skills; 2) What challenges occurred during program implementation and were these able to be overcome? The multidisciplinary evaluation team consisted of physicians and staff skilled in quality improvement methodologies, mixed and qualitative research methods, programs evaluation, and statistics. Consistent with common use of mixed-methods in dissemination and implementation science [32], for our first question we used a quantitative method, a longitudinal survey, to examine deductively the outcomes of the implementation with breadth across team member participants. For our second question, we employed multiple qualitative methods, individual and group interviews/discussions, to examine inductively with depth the implementation context and process by sampling multiple participants and implementers during the first two cohorts (when significant challenges were expected to be discovered), and program leaders across the entire implementation period. Individual interviews were used for participants to enhance confidentiality, and group interviews were used for implementers and leaders in order to capture the range of opinions and agreement. These concurrent methods received equal weight (QUAN + QUAL) and were mixed for complementarity. Throughout the study period because our research was closely embedded within the implementation process, we used an empowerment evaluation approach [33], which aims to aid organizations in improving their innovation during the implementation process through sharing and discussing data and encouraging reflection. Therefore, during the program we collected and reported to program leadership findings about challenges from the qualitative data near “real time” and reported the analyzed quantitative outcome data every 6 months. As described further below, the summative evaluation in this paper presents the quantitative longitudinal outcomes, context and resolved and ongoing challenges analyzed from the qualitative data across the multi-year program.

### Data collection

Quantitative evaluation consisted of an electronic survey sent to all team member participants at baseline, 6, and 12 months. Across all cohorts and time periods, 15 items assessed perceptions in the following domains: organizational support, team cohesion and effectiveness, quality improvement and meeting skills, and communication effectiveness. A single question assessed overall satisfaction with the program at 6 and 12 months. Responses were generally on a five-item Likert scale (strongly agree, agree, neutral, disagree, strongly disagree). Two survey items about patient engagement were analyzed for a separate publication [34] and thus these findings are not presented. Several survey items were taken from prior Microsystems tools [28, 35] while others were created or adapted for the local context. These adaptations reflected the local purpose or specific leadership roles and structures (e.g. “to make my work meaningful” adapted to “to be effective in my job”; “clinic manager support”; “physician leader communication”).

These data were augmented by individual interviews, focus groups and facilitated stakeholder group discussions led by the first author or a masters-level researcher trained in qualitative methods. Interviews were generally conducted on-site and lasted 15–75 min. During the first and second cohort (2009–2010), 69 team members from 11/17 teams were interviewed. Program leaders informed the selection of these teams using a maximum variation approach by varying primary care specialty, location (rural or urban), and degree of engagement in program activities. All team members who were present on the days that the research team was on-site were interviewed. Because teams microsystems varied in membership, interviewees came from a variety of roles: 14 physicians (3 residents), 6 Advanced Practice Providers, 8 Administrative supervisors, 1 Pharmacist, 7 Registered or Licensed Practical Nurses, 17 Medical Assistants/Certified Nursing Assistants, 4 Schedulers, 4 Radiology, Laboratory or Medical Technicians, and 8 Receptionists. Additionally, two one-hour clinic manager and program leader focus groups and one coach focus group were held. Lastly, four longitudinal, facilitated structured discussions about ongoing lessons learned were held with stakeholders responsible for program development, implementation, and evaluation. Six participants requested that their interview not be recorded. In these cases, handwritten notes were taken during the interview and narrative summaries were constructed immediately afterwards. For the remaining 63 interviews, all focus groups, and structured discussions, audiotaping and transcription occurred.

### Data analysis

Survey data were analyzed with STATA version 14 software (StataCorp, College Station, Texas) employing ordinal logistic regression adjusting for role (Physician/APP/Pharmacist, nurse, medical assistant, receptionist, other) with time as an indicator variable and a robust estimate of the variance that accounted for clustering by clinic and cohort. Analyses compared individual aggregate responses at 6-months and 12-months to those at the baseline period and were considered significant at *p* < 0.05. Missing data, ranging from 3 to 5% per question, were dropped after examination revealed no systematic pattern.

Qualitative analyses employed an immersion/crystallization approach [36]. Two individuals skilled in qualitative analysis independently read all transcripts and summaries, understanding each clinic team as a case and paying particular attention to any text that identified implementation challenges and solutions. This text was transferred to a structured matrix in Excel that was organized by both clinic site, cohort, and role. Text was examined line by line and coded to identify the type of challenge (e.g. time, knowledge, technology, motivation, spread) and attempted solution. These codes were aggregated into themes by topic (e.g staffing and support, participation motivators, sustainment), and the researchers met weekly to discuss these themes. Because of the participatory aspect of the evaluation, researchers also triangulated quantitative findings with the qualitative themes that emerged across the different levels of data by role (individual team members, managers, coaches, and program leadership). This allowed program implementers to make adaptations to the intervention and involve those that were experiencing the challenge. Member checking occurred through regular public presentation of findings back to participating individuals and leadership as part of the participatory approach to the evaluation.

## Results

Table 2 provides examples of successful team projects by specialty.

**Table 2:** Microsystem team project examples

Specialty	Project
Pediatrics	● Increase percentage of time immunizations are done before MD enters visit so patient/family wait time is decreased ● Increase percentage of patients ages 11–18 who have a biannual well child check ● Complete chart review and pending orders by rooming staff prior to MD entering clinical encounter
Family Medicine	● Reduce the number of refill calls/requests after a visitby creating a process to ask if refills are needed at every visit and documenting if refills were reviewed ● Increase the percentage of patients seen for acute visits ● Improve timeliness of visits through decreasing the amount of time between scheduled visit time and provider log in time
General Internal Medicine	● Increase the percentage of after visit summaries given to patients that have provider instructions ● Increase the number of scheduled chronic care follow-up appointments ● Increase the number of patients with activated MyChart accounts (electronic health record patient portal)

### Survey results

204/257 (79%) of individuals completed the baseline survey, with responses from all 58 teams. Across cohorts of teams receiving Microsystems training, response rates ranged from 49 to 92% at baseline, 45–77% at 6 months, and 52–81% at 12 months. The mean (standard deviation) response to the question “participating in the Microsystems program has been a worthwhile experience” was 3.83 (0.91) at 6 months and 3.80 (0.90) at 12 months, with responses on a scale of 5 = strongly agree, 4 = agree, 3 = neutral, 2 = disagree, 1 = strongly disagree.

Table 3 displays longitudinal aggregate mean responses to the remaining questions organized by domain. In terms of organizational support, respondents did not perceive a significant change in their clinic manager’s support of their microsystems work (4.10 (mean), baseline (time point); 4.21 6 months; 4.25, 12 months). At 12 months compared to baseline, there were statistically significant increases in microsystem team member’s perceptions of having the tools and equipment needed to be effective (3.32, baseline; 3.50, 6 months; 3.66, 12 months) and useful knowledge and information about their patient population (3.38, baseline; 3.69, 6 months; 3.74, 12 months).

**Table 3:** Aggregate mean response to survey questions at baseline, 6-months, and 12-months

	Baseline		6-months		Significance* (*p*-value)	12-months		Significance* (*p*-value)
Domain and question	N	Mean (SD)	N	Mean (SD)		N	Mean (SD)	
Organizational support								
I am given all the tools and equipment I need to be effective in my job.	200	3.32 (0.98)	157	3.50 (0.95)	0.358	129	**3.66 (0.83)**	**0.013**
I have useful knowledge and information about our patient population (e.g., demographics, clinical outcomes, satisfaction, what they want and need).	200	3.38 (0.96)	156	3.69 (0.74)	0.081	124	**3.74 (0.78)**	**0.007**
My clinical microsystem work is supported by our clinic manager.	204	4.10 (0.77)	156	4.21 (0.82)	0.244	130	4.25 (0.73)	0.281
Team effectiveness and cohesion								
I feel confident about the capability of my group to perform the tasks very well.	203	4.06 (0.74)	156	4.09 (0.64)	0.744	125	4.07 (0.65)	0.912
I feel confident that my group will be able to manage effectively unexpected troubles.	203	3.95 (0.78)	154	4.12 (0.58)	0.100	125	4.09 (0.67)	0.419
My group is able to solve difficult tasks if we invest the necessary effort.	202	3.94 (0.81)	156	**4.19 (0.52)**	**0.014**	125	4.15 (0.60)	0.194
My microsystem team is fully competent to make team level improvements.	201	4.00 (0.75)	156	4.15 (0.58)	0.143	125	4.14 (0.59)	0.324
I have a strong bond with most of the members of my microsystem team.	203	3.90 (0.78)	156	**4.16 (0.70)**	**0.017**	125	4.16 (0.68)	0.055
Meeting and quality improvement skills								
I know the top processes that need to be improved and are known to be a source of patient complaints.	199	2.58 (0.87)	156	**3.81 (0.72)**	**<.0001**	123	**3.83 (0.67)**	**<.0001**
I know how to use data to identify opportunities for improvement.	201	3.08 (0.93)	156	**3.95 (0.61)**	**<.0001**	124	**4.00 (0.60)**	**<.0001**
I can create a meeting agenda that keeps us focused on specific improvement aims.	201	3.03 (0.88)	155	**4.03 (0.63)**	**<.0001**	124	**4.06 (0.72)**	**<.0001**
I can play the role of facilitator, meeting leader, timekeeper or recorder, as needed.	202	3.30 (0.93)	156	**4.18 (0.60)**	**<.0001**	123	**4.02 (0.84)**	**<.0001**
I know the purpose of our microsystem team.	201	3.22 (0.94)	158	**4.10 (0.72)**	**<.0001**	130	**4.08 (0.75)**	**<.0001**
Team communication effectiveness								
I feel our team has an effective communication plan for keeping the physician clinic leader (e.g., clinic medical director or site lead) informed of our microsystem work.	196	3.13 (0.89)	156	**4.01 (0.74)**	**<.0001**	124	**4.00 (0.79)**	**<.0001**
I contribute and share my team’s improvements with other teams in the practice; we have an effective communication plan for keeping others informed.	197	2.96 (0.91)	156	**3.48 (0.93)**	**<.0001**	123	**3.40 (0.96)**	**0.043**

*Bold indicates significance at *p* < 0.05

Respondents perceived high team effectiveness and cohesion from the outset of the program. Two items showed statistically significant improvements at 6 months—the ability to solve difficult tasks if effort was invested (3.94, baseline; 4.19, 6 months) and having a strong bond with team members (3.90, baseline; 4.16, 6 months). Items that did not show significant changes queried about group capability to perform tasks well, manage unexpected troubles, and competency to make team level improvements.

Also as shown in Table 3, responses indicate that team members perceived significant gains over baseline at 6 months and 12 months in all five quality improvement skills queried: knowing the top processes needing improvement, using data to identify improvement activities, creating a focused meeting agenda, serving in different team roles and knowing the purpose of the team. Respondents also perceived significant gains in effectively communicating their work to the manager (3.13, baseline; 4.01, 6 months; 4.00, 12 months) and other teams in the practice (2.96, baseline; 3.48, 6 months; 3.40, 12 months).

### Qualitative results

Several key challenges to the success of the program were identified by teams and clinic leadership during the implementation process. For many of these, the organization was able to respond to Microsystem program needs and develop specific solutions; Table 4 describes challenges where a solution was implemented, along with a representative quote. These included: lack of time and coverage for program participation, need for technical/technology support, lack of perceived value for improvement work, and difficulty spreading learnings.

**Table 4:** Challenges requiring solutions during Microsystem Implementation

Challenge	Representative quote	Solution implemented
Lack of time and coverage for participation	*“We are a busy clinic, we see a lot of patients. It’s hard to take on—don’t get me wrong, it’s not hard to take on new stuff, it’s nice to see the change, but it’s really hard to implement it if you don’t have the time to do it.”* *“The other receptionists worried they would have to carry my load if I’m away from the phone. Most of the time, we have to have coverage for me…and I didn’t want them to think they had to carry my load if I’m not there.”*	▪ Designated protected time during work hours for team meetings ▪ Float coverage provided for learning sessions
Need for Electronic Health Record technical support to implement certain interventions	*“So we should be using this tool [the EHR], and we have this tool, and they started this microsystems process saying you absolutely may not use that tool. That’s nuts. That’s ludicrous. You do not want people thinking that way. You want people to be recognizing that that’s a tool to use.”*	▪ 1.8 full-time equivalents of dedicated electronic health record consultation were hired for teams’ support
Lack of team member’s computer skills, technology and training	*“I am really slow at compiling my notes. If I’m the recorder for the minutes, I’m really slow. So if I’m doing that, that’s a good half a day extra.”*	▪ Provided teleconference, computer equipment and training
Improvement work not perceived as being valued	*“Its research that’s rewarded, teaching is important, and how much, how many dollars you bring in. But there’s no reward or clout for administrative skills”*	▪ Provided physician continuing medical education credits and medical assistant continuing education credits (American Association of Medical Assistants approved) for learning session participation ▪ Space made available within the clinic for team meetings and data displays
Dissemination to other teams difficult because of varied project focuses	*“I can’t imagine learning anything from other clinics. Every clinic is so different”*	▪ Teams directed to align projects to focus on access and efficiency
Opportunities to share improvement work	*“So far it’s just been kind of osmosis, some of the things. The entire staff we send our minutes to and we have a bulletin board in the back. So we have it posted and we try to be transparent, but I think it’s ineffective.”*	▪ Hosted an annual summit for improvement sharing and collaboration ▪ Created “Improvement Solutions” searchable web site for completed improvement projects from across organization

However, additional challenges persisted. These challenges are described below including: tensions between team-and clinic-level change, part-time workforce, team instability, challenge of a structured QI approach, and difficulties aggregating learnings.

#### Tensions between team and clinic level change

Coaches, who could structure meetings and teach improvement skills, lacked the operational authority of clinic managers who are responsible for daily operations and clinic-wide performance. These differing positions led to conflicts, particularly when a team’s improvements were viewed as too disruptive to existing clinic workflow or culture. Additionally, for the first two cohorts, leadership discouraged clinic managers from being present at team meetings out of concerns that this would decrease front-line quality improvement learning and innovation. This independence was a major shift in culture. As one manager explained: “*I think the more uniformity you have, the more people can adhere to what the expectations are rather than having one group doing this and one group doing that- and then trying to manage that…is really difficult*.”

#### Part-time workforce

The Microsystem approach was designed for single physician teams who engaged in accomplishing QI goals throughout the week in between weekly meetings. However, in this organization many of the clinicians and staff were part-time. This created difficulty in figuring out how to equitably divide the between-meeting work. It was also difficult to schedule weekly meetings so that the entire team could attend. As a medical assistant explained, “*Four of us are part-time workers so it means that every week somebody comes in on their day off to work on microsystems*.” Lastly, situations arose where a part-time staff member received training as part of one team, and then was also part of a team that in a future training cohort. It was unclear if and how much of the training should be repeated in this circumstance.

#### Team instability

Turnover occurred particularly within certain positions (e.g., medical assistants). Their replacements were added to the team because it made practical sense given that they were part of the natural microsystem. However, bringing on someone new was perceived as difficult because there was no one clearly responsible for on-boarding, and understanding the program required time. As a team member explained, *“When you do have a new employee training for Microsystems we can take time out of our team meeting to train, but we’re not as productive and that puts somebody on the spot-- the new trainee.”* Also, some physicians changed their clinical FTE during the program. Residents additionally had variables schedules. This led to shifts and difficulties in scheduling team meetings and/or variable attendance at meetings.

#### Implementing a data-driven QI approach

The Microsystems approach required teams to spend time evaluating their microsystem in a data-driven process and learning a standard language and set of QI tools. Teams could not select improvement foci based solely on perceptions of what should be addressed-this gap had to be demonstrated with data. This process felt cumbersome to several team members because collected data did not always capture the ‘stories’ that impact individual Microsystems. As one put it, “*You need something for people to grab on to, feel some success with and then maybe you can understand the process better. Just going in and trying to understand the process and then picking something to do [is] harder to do than having some ideas and seeing if you can’t fit them into the process.”*

#### Difficulty aggregating learnings

Despite the directive to teams to focus on organizational improvement priorities (e.g., access), variations in clinic structure, specialty, and culture made it difficult to spread successful improvement practices. Teams that successfully improved quality documented their approaches in a “playbook.” However, there was not a standard mechanism or clear accountability for spreading these playbooks to teams at the same delivery site or to other clinics in the organization. Operational leaders concluded that the process of disseminating Microsystem-discovered improvements system-wide was “*too long and costly.*” In contrast, the organization was achieving significant gains in quality measures using a “top down” approach: designing improved processes and structures centrally, testing them with Microsystems trained teams, and spreading these improved processes across clinics with local leadership support [28]. As one leader pointed out, “*Developing the Microsystems program was not sufficient in and of itself to achieve organizational transformation….we had to address all levels of change, not just the patient and care team*.”

### Integrating quantitative and qualitative results

From the survey findings, team members perceived the program provided them with additional support- data, knowledge, equipment and improved communication. The in-depth interview findings did not contradict these findings. However, leaders did not measure the success of the program according to these metrics. Instead, the focus was on resource use, rapidity of change, and scalability.

## Discussion

This paper describes and evaluates the multi-year experience of a health system that implemented a Microsystem approach to primary care redesign in pursuit of the quadruple aim. The program significantly increased perceived QI skills and bonds between team members. However, it required adjustments in response to implementation barriers such as the need for dedicated time, coverage, and technical support. Significant challenges persisted due to the nature of primary care in an academic health center including part-time clinicians, turnover, and competing responsibilities.

Our findings suggest important considerations for health systems that are embarking on systematic approaches to redesign in pursuit of high value care. Similar to existing literature [20, 21] we found the microsystems approach improved relationships and clarity of purpose at the team level. However, the resources required to implement, maintain, and disseminate the program were formidable. Although implemented in a setting dedicated to holistic change across primary care disciplines [22], this approach alone was insufficient to achieve transformative primary care redesign in a time frame that was acceptable to the organization.

Operational leaders need to balance standardization of medical best practices with the variation present across clinics within a health system. Doing so requires stakeholder input, and the Microsystem approach may promote buy-in with front line clinicians and staff. Implementation of this program also did lead to important information for leadership as it continued to embark on primary care redesign. Variation in team processes, culture, structure, skills and knowledge were uncovered through this program. For example, variation across the system (e.g., union versus non-union staff, years using an electronic health record (EHR)) contributed to clinic-to-clinic variation in the Microsystem experience. Insights into these variations provided critical input to future work that standardized primary care team composition and care models [23], developed a training program for clinic dyad leaders (physicians and managers), and focused on system-level supports (e.g., optimal use of EHR [37], enterprise-wide care guidelines).

The health system’s Microsystem program was ended and replaced by a centralized QI approach that simultaneously addressed change at all levels of the health system, including but not exclusively focused on the microsystem level. With the prior focus on the microsystem, teams were not empowered to make changes at the organizational level. They could only improve processes within the constraints of existing system-wide structures such as EHR functionality or centralized calling centers. In the shift to a more centralized approach, multi-disciplinary teams with meso- and macro-leaders were able to improve infrastructure which ‘made it easy’ for microsystem teams to achieve improvements in care. Primary care initiatives continued to be piloted with teams that had received microsystems training. These teams had achieved a cultural shift and understood the need for data-driven change. They also had regular practice in using quality improvement skills. They were able to apply their testing skills to inform the design of final system standards [28]. As a consequence of these whole-system redesign efforts directed at improving preventive and chronic care, teams were able to achieve significant gains in publicly-reported primary care performance metrics across primary care clinics. Implications of our findings for others both in the United States and elsewhere is the critical importance of providing a clear process of quality improvement education across all stakeholder levels – leadership, operational management, and teams – in order to increase engagement in the change process, improve communications and clarity of purpose, and develop a culture that is able to improve outcomes. One increasingly popular approach in primary care is the Lean management system, which can empower staff at all levels, promote teamwork [38], and lead to an innovation culture [39]. Recent studies of factors leading successful implementation of the Lean approach reveal the crucial role of leadership [40, 41], also suggesting a different entry point than the microsystems level in the quest for transformative change.

Our evaluation is subject to several strengths and limitations that should be taken into account when examining these findings. Strengths include relatively high participation rates in data collection over multiple time points, a multi-year implementation across several teams, and extensive qualitative data collection employing multiple methods and frequent member checking that increased suggestion of the validity of our results. Limitations include the transferability of results from practices affiliated with a Midwestern Academic Health Center, the focus on evaluation of the implementation rather than clinical outcomes, missing data from survey or item nonresponse over time, the multiplicity effect of assessing multiple outcomes, and use of aggregate rather than individually linked responses. Future research should consider evaluating differences and similarities in the implementation and sustainability of primary care practice quality improvement interventions across different practice settings and sizes. However, our implementation study in an academic setting with education, research and clinical missions is likely representative of other settings where multiple priorities compete for limited resources. Similar to other studies [21], our experience identified important limitations to implementing clinical microsystems which may limit the feasibility of this method in widespread primary care redesign efforts.

## Conclusion

These findings suggest that a microsystems approach is valuable for building team relationships and quality improvement skills but is challenged in a large, diverse academic primary care context. Transformative change requires purposeful redesign across the entire system [42]. Microsystem investment alone may facilitate change but is not sufficient to respond to the challenges of a rapidly changing health care environment and the need to compete in value-focused markets.

## Abbreviations

EHR: Electronic health record
QI: Quality improvement

## References

[1] Washington AE, Coye MJ, Feinberg DT (2013). Academic health centers and the evolution of the health care system. JAMA.

[2] Dzau VJ, Cho A, ElLaissi W, Yoediono Z, Sangvai D, Shah B (2013). Transforming academic health centers for an uncertain future. N Engl J Med.

[3] Miller HD (2015). Making Value-Based Payment Work for Academic Health Centers. Acad Med.

[4] Nelson EC, Batalden PB, Huber TP, Mohr JJ, Godfrey MM, Headrick LA (2002). Microsystems in health care: part 1. Learning from high-performing front-line clinical units. Jt Comm J Qual Improv.

[5] Quinn JB (1992). Intelligent Enterprise: a knowledge and service based paradigm for industry.

[6] The Dartmouth Institute | Microsystem Academy. http://www.clinicalmicrosystem.org/. Accessed 19 Sep 2018.

[7] Anderson D, Khatri K, Blankson M (2015). Using clinical microsystems to implement care coordination in primary care. J Nursing Care.

[8] O’Dwyer C. The Introduction of Clinical Microsystems into an Emergency Department. 2014. p. 126.

[9] Tess AV, Yang JJ, Smith CC, Fawcett CM, Bates CK, Reynolds EE (2009). Combining clinical microsystems and an experiential quality improvement curriculum to improve residency education in internal medicine. Acad Med.

[10] von Plessen C, Aslaksen A (2005). Improving the quality of palliative care for ambulatory patients with lung cancer. BMJ.

[11] Bobb Jennifer, Lee Amy, Lapham Gwen, Oliver Malia, Ludman Evette, Achtmeyer Carol, Parrish Rebecca, Caldeiro Ryan, Lozano Paula, Richards Julie, Bradley Katharine (2017). Evaluation of a Pilot Implementation to Integrate Alcohol-Related Care within Primary Care. International Journal of Environmental Research and Public Health.

[12] NHS England » General Practice Forward View. https://www.england.nhs.uk/gp/gpfv/. Accessed 19 Sep 2018.

[13] Union PO of the E (2018). A new drive for primary care in Europe : rethinking the assessment tools and methodologies : report of the expert group on health systems performance assessment.

[14] Breton Mylaine, Smithman Mélanie Ann, Touati Nassera, Boivin Antoine, Loignon Christine, Dubois Carl-Ardy, Nour Kareen, Lamoureux-Lamarche Catherine, Brousselle Astrid (2018). Family Physicians Attaching New Patients From Centralized Waiting Lists: A Cross-Sectional Study. Journal of Primary Care & Community Health.

[15] van Weel C, Kassai R. Expanding primary care in south and East Asia. BMJ. 2017;356:j634. 10.1136/bmj.j634 28242604

[16] Mossman K, Onil Bhattacharyya MD, McGahan A, Mitchell W. Expanding the reach of primary Care in Developing Countries. Harv Bus Rev. 2017; https://hbr.org/2017/06/expanding-the-reach-of-primary-care-in-developing-countries. Accessed 19 Sep 2018.

[17] Wagner EH, Austin BT, Von Korff M. Improving outcomes in chronic illness. Manag Care Q. 1996;4:12–25. 10157259

[18] Berwick D, Kilo C (1999). Idealized design of clinical office practice: an interview with Donald Berwick and Charles kilo of the Institute for Healthcare Improvement. Manag Care Q.

[19] Kilo CM, Wasson JH (2010). Practice redesign and the patient-centered medical home: history, promises, and challenges. Health Aff.

[20] Kollisch DO, Hammond CS, Thompson E, Strickler J (2011). Improving family medicine in Kosovo with microsystems. J Am Board Fam Med.

[21] Williams I, Dickinson H, Robinson S, Allen C (2009). Clinical microsystems and the NHS: a sustainable method for improvement?. J Health Organ Manag.

[22] Carney PA, Eiff MP, Green LA, Carraccio C, Smith DG, Pugno PA (2015). Transforming primary care residency training: a collaborative faculty development initiative among family medicine, internal medicine, and pediatric residencies. Acad Med.

[23] Koslov S, Trowbridge E, Kamnetz S, Kraft S, Grossman J, Pandhi N (2016). Across the divide: “primary care departments working together to redesign care to achieve the triple aim.”. Healthcare.

[24] Bodenheimer T, Sinsky C (2014). From triple to quadruple aim: Care of the Patient Requires Care of the provider. Ann Fam Med.

[25] Kotter JP. Leading change: why transformation efforts fail. Harv Bus Rev. 1995;73(2):59–67.

[26] Institute of Medicine (US) Committee on Quality of Health Care in America (2001). Crossing the Quality Chasm: A New Health System for the 21st Century.

[27] Shortell S, Kalunzy A. Organization theory and health services management. In: Health care management: organization design and behavior. 4th ed. Delmar: Cengage Learning; 2011.

[28] Kraft S, Carayon P, Weiss J, Pandhi N (2015). A simple framework for complex system improvement. Am J Med Qual.

[29] Institute for Healthcare Improvement: How to Improve. http://www.ihi.org:80/resources/Pages/HowtoImprove/default.aspx. Accessed 19 Sep 2018.

[30] Heuvel JVD, Does RJMM, Koning HD (2006). Lean six sigma in a hospital. Int J Six Sigma Competitive Advantage.

[31] Lane DC, Oliva R (1998). The greater whole: towards a synthesis of system dynamics and soft systems methodology. Eur J Oper Res.

[32] Brownson RC, Colditz GA, Proctor EK (2012). Editors. Dissemination and implementation research in health: translating science to practice. 1 edition. Oxford.

[33] Fetterman DM (1994). Empowerment Evaluation. Am J Eval.

[34] Davis S, Berkson S, Gaines ME, Prajapati P, Schwab W, Pandhi N (2016). Implementation science workshop: engaging patients in team-based practice redesign — critical reflections on program design. J Gen Intern Med.

[35] Reason P, Bradbury-Huang H, editors. The SAGE handbook of action research: participative inquiry and practice. Second ed. London: SAGE publications Ltd; 2013.

[36] Crabtree BF, Miller WL (1999). Doing qualitative research.

[37] Pandhi N, Yang W-L, Karp Z, Young A, Beasley JW, Kraft S (2014). Approaches and challenges to optimising primary care teams’ electronic health record usage. J Innovation in Health Informatics.

[38] Hung DY, Harrison MI, Truong Q, Du X (2018). Experiences of primary care physicians and staff following lean workflow redesign. BMC Health Serv Res.

[39] Henrique DB, Filho MG (2018). A systematic literature review of empirical research in lean and six sigma in healthcare. Total Qual Manag Bus Excell.

[40] Laureani A, Antony J (2018). Leadership – a critical success factor for the effective implementation of lean six sigma. Total Qual Manag Bus Excell.

[41] van Assen MF (2018). Exploring the impact of higher management’s leadership styles on lean management. Total Qual Manag Bus Excell.

[42] Berwick DM (2002). A User’s manual for the IOM’s ‘quality chasm’ report. Health Aff.

